# Sex and Age Differences in Exposure to Secondhand Smoke at Home among Korean Adolescents: A Nationally Representative Survey

**DOI:** 10.3390/ijerph13020241

**Published:** 2016-02-19

**Authors:** Jun Hyun Hwang, Soon-Woo Park

**Affiliations:** Department of Preventive Medicine, Catholic University of Daegu School of Medicine, 33 Duryugongwon-ro 17-gil, Nam-gu, Daegu 42472, Korea; pmdr213@cu.ac.kr

**Keywords:** secondhand smoke, environmental tobacco smoke, age, sex, vulnerable populations, adolescents

## Abstract

The authors assessed sex and age differences in secondhand smoke (SHS) exposure among vulnerable adolescent populations. Data from the 2013 Korea Youth Risk Behavior Web-based Survey of 64,499 non-smokers aged 13–18 years were analyzed using multiple logistic regression. Girls were exposed 1.26 times (95% confidence interval, 1.21–1.32) more to home SHS than boys, and the younger adolescents were more likely to be exposed to home SHS than were the older, regardless of sex (*p* < 0.001). Younger girls living with or without current smokers and the younger boys living with current smokers were more likely to be exposed to SHS at home, when the data were stratified according to current household member smoking, which was one of the main risk factors for SHS exposure at home. Girls living with current smokers were more likely to be exposed to SHS at home than boys regardless age. Girls and younger adolescents, populations vulnerable to smoke exposure, were more likely to be exposed to SHS at home, even though they should be more protected. It is necessary to improve home SHS awareness, especially among these vulnerable populations.

## 1. Introduction

People can be exposed to secondhand smoke (SHS) in various places including homes, vehicles, workplaces, and public places. Long-term SHS causes heart disease and lung cancer, as well as other adverse health outcomes in adolescents such as acute respiratory infection, ear problems, worsening of asthma, and decreased pulmonary growth velocity [1]. In addition, smoking initiation risk increases when non-smoking adolescents are exposed to SHS [2].

Since about 30% of adolescents worldwide who have never smoked are exposed to SHS at home, it is reasonable to assume that home is where adolescents are exposed to SHS most frequently [3]. As home SHS exposure is especially difficult to avoid in the presence of household smokers, home is one place where adolescent SHS exposure needs to be urgently addressed.

There have been many studies on the risk factors for adolescent home SHS. In a systematic review of 41 studies of home SHS exposure in children under 18 years old published before July 2014, Orton *et al.* analyzed risk factors for home SHS in five categories: (1) socioeconomic status (SES); (2) parental characteristics (education, age, race); (3) family and home characteristics (family size, family structure, home environment); (4) children’s characteristics (age, gender); and (5) parental smoking characteristics (smoking behavior, attitudes) [4].

Among the various biological risk factors, females and younger adolescents are more vulnerable to SHS for the following reasons. The lung could be adversely affected by SHS exposure due to immaturity at a younger age. As pulmonary function continuously grows until late teens, SHS exposure in this period could affect lung maturation [5,6]. Especially, girls are more vulnerable to the effects of even a very small amount of smoking on pulmonary function growth [7,8,9]. In addition, smoking initiation risk for children 12–18 years of age increases an average of 10% per year [10]. Considering that SHS exposure affects adolescent smoking initiation [2], it is important to evaluate the level of home SHS experiences among middle and high school students according to different age groups to protect from smoking.

In their systematic review of 41 studies, Orton *et al.* [4] examined 19 reports of gender-specific and 19 reports of age-specific effects on children under 18 years old. Only six of the 19 studies included adolescents over 15 years of age as study subjects [11,12,13,14,15,16], among which home SHS exposure increased with age in three studies but decreased in only one [11]. Among the six studies on mid- and late-teen adolescents [11,12,13,14,15,17], girls were more exposed to SHS at home in only two [11,13]. Thus, adolescent home SHS exposure level according to age and gender shows contrasting results, and not enough evidence concerning this issue has yet accumulated. Furthermore, most existing studies were conducted in the West, with only two being conducted in Asian countries; namely, India and Mongolia. SHS exposure increased along with age in the Mongolian study, and it was higher in males. However, there were no statistically significant gender differences in either the Indian or the Mongolian studies [14,16].

According to the Korea Youth Risk Behavior Web-based Survey (KYRBS), a representative health behavior survey of 7th–12th grade Korean adolescents [18], SHS exposure rate at home for one day or more per week was consistently higher among middle than among high school students from 2007 to 2014. In addition, it was consistently higher in females than males, which was different from the results of the existing Asian studies. The strongest risk factor of home SHS is the presence of smokers in the family, among which parents’ smoking has the most influence [4]. However, the smoking rate of Korean adult males in 2013 was 42.1%, while that of females was only 6.2% [19]. Whereas the daily smoking rate of adults is higher than the average of OECD nations, that of adult females is very low, making Korea one of the nations with a highly differential gender-specific smoking rate [20]. Therefore, considering adolescents come most in contact with their mothers at home, the large smoking rate gender gap may lead to a differential result by exerting a significant influence on home SHS exposure.

Thus, the present study was conducted with non-smoking Korean adolescents (middle and high school) in order to assess whether biologically vulnerable population, girls or younger students, were relatively protected from home SHS exposure.

## 2. Materials and Methods

### 2.1. Study Population

We analyzed data from the 10th Korea Youth Risk Behavior Web-based Survey (KYRBS-X) conducted in 2014 by the Korea Centers for Disease Control and Prevention. The KYRBS is a nationally representative, self-reported, and anonymous online survey of Korean students enrolled in grades 7 through 12 (middle and high school students, ages 13–18). The KYRBS uses a stratified multistage probability sampling design to produce nationally representative statistics on Korean adolescent health behaviors. A total of 72,060 students from 799 schools (400 middle and 399 high schools) completed the KYRBS-X (response rate = 97.2%) [18]. After excluding current smokers and students with missing data concerning home SHS exposure, 64,499 non-smokers (30,925 boys and 33,574 girls) remained in the study population. Here, non-smokers are defined as adolescents who reported not having smoked in the past month. This secondary data analysis was approved as exempt from review by the Institutional Review Board of the Daegu Catholic University Medical Center (CR-15-084).

### 2.2. Measures

According to the traditional definition of World Health Organization, SHS is defined as “the combination of smoke emitted from the burning end of a cigarette or other tobacco products and smoke exhaled by the smoker” [21]. However, the concept of thirdhand smoke, “residual tobacco smoke pollutants that remain on surfaces and in dust after tobacco has been smoked”, has emerged and was differentiated from SHS recently [22]. In this new perspective, the term “SHS” could be considered as not synonym but one part of passive smoking or Environmental tobacco smoke [23].

In this study, we focused on exposure to SHS from the traditional viewpoint with exception of thirdhand smoke. Therefore, the home SHS outcome variable was evaluated using the following question: “During the past 7 days, on how many days have people smoked in your home, in your presence?” Exposure to home SHS was defined as having had people smoke in one’s presence on one or more days within the past 7. The main independent variables were sex and grade, which was used as a surrogate indicator of age. If more than one of the students’ family members, including father, mother, siblings, grandparents, or others currently smoked cigarettes, they were classified as having a currently smoking household member.

Closest friend smoking was assessed using the responses to the following question: “Do any of your closest friends smoke tobacco?” The participants were provided with four possible answers: (1) None of them; (2) Some of them; (3) Most of them; and (4) All of them. Here, since the sample size of the “all of them” group (0.8%) was too small to be separately categorized, this group and the “most of them” group (5.0%) were combined (most/all). The other covariates included region of residence (metropolitan city, city, or province) and perceived economic status (high, middle, or low). The original Korean version of core questionnaires is available in Supplementary Materials Table S1.

### 2.3. Statistical Analysis

Multivariate logistic regression was conducted to estimate the relationship between home SHS and sex and grade after adjusting for covariates including location, perceived economic status, closest friend smoking, and current household member smoking. Since current household member smoking is closely associated with home SHS, w model based on this variable was used to estimate the effect of sex and grade on home SHS. All analyses were performed using SPSS version 19.0 (IBM: Armonk, NY, USA), and a *p*-value of <0.05 was considered significant. All results are presented following complex SPSS sampling procedures to represent the Korean adolescent population.

## 3. Results

Among adolescent non-smokers (*N* = 64,499), 49.3% and 50.7% of them were boys and girls, respectively. Prevalence of home SHS was higher among girls (34.1%) than boys (31.1%), and it increased with decreased grade, regardless of sex. Students having a household member who currently smoked cigarettes were 5.6 times more likely to be exposed to home SHS than those without currently smoking household members (Table 1).

After adjusting for all the covariates, current household member smoking was the most powerful home SHS risk factor among all variables, having an adjusted odds ratio (OR) value of 9.98 (95% confidence interval (CI), 9.51–10.47). Girls were 1.26 times (95% CI, 1.21–1.32) more exposed to home SHS than boys, and the lower grade students was more likely to be exposed to SHS at home than the higher grade students, regardless of sex (*p* < 0.001) (Table 2).

Stratification models according to current household member smoking are shown in Table 3 and Table 4. Among girls, home SHS risk significantly increased along with decreasing grade (*p* < 0.001) from 1.01 (95% CI, 0.84–1.21) for the 11th grade to 1.33 (95% CI, 1.07–1.66) for the 7th grade for those without current household smokers, and from 1.03 (95% CI, 0.94–1.13) for the 11th grade to 1.46 (95% CI, 1.31–1.62) for the 7th grade for those with them. On the other hand, among boys, these increasing patterns of home SHS risk according to grade were not observed among students in all grades without currently smoking household members. However, lower grade boys were more likely to be exposed to home SHS with consistency between 7th and 10th grades, and 7th grade boys had the highest home SHS exposure risk among all middle and high school boys (Table 3).

Among students who did not live with current smokers, there was no significant association between home SHS exposure and sex in both the total and separate grades stratified subgroup analysis, except for the 10th grade. However, among students living with current smokers, girls were consistently (1.29 to 1.52 times) more likely to be exposed to home SHS than boys, regardless of grade (Table 4).

## 4. Discussion

Home SHS exposure increased along with decreasing age, regardless of whether smokers were present in the home, and females experienced more exposure than did males when there was a smoker in the home. According to the 2013 Korea National Health and Nutrition Examination Survey, the smoking rate of adult males, the main source of Korean home SHS, is continuously decreasing, and the SHS exposure rate of non-smoking adult females also decreased sharply from 23.9% in 2005 to 13.7% in 2013 [19]. However, according to KYRBS, over 30% of non-smoking adolescents are exposed to home SHS, which is a much higher rate than that of adults, and the decreasing trend from 40.3% in 2006 to 33.8% in 2014 is not as sharp [18]. Although the adult and adolescent results were calculated from two different surveys, both are reliable because these were the national surveys representing Korean adults and adolescents, respectively. In addition, the fact that the decline depth witnessed among the adolescents is much shallower when the home SHS exposure trends are compared suggests that the vulnerable adolescent group is not well protected against SHS.

The home SHS exposure trend among Korean adolescents can be explained by their sociocultural backgrounds. First, Korean high school students (10th–12th grades) are dismissed from school at a later time than are middle school students (7th–9th grade). High school students have less of a chance to be exposed to home SHS because they have less contact with their parents. They do not usually stay home too long due to classes and university entrance examination preparation.

Second, the smoking rate of Korean adult males is highest among those in their 30s and declines afterwards. The smoking rates of those in their 30s, 40s, and 50s, which correspond to the age group of the fathers of the middle and high school students, are 54.5%, 48.0%, and 40.8%, respectively, thus decreasing substantially with the increase in age. Therefore, since parents of lower grade students are relatively younger, the smoking rate of fathers of lower grade students is estimated to be higher than that of fathers of higher grade students, which supports the notion that home SHS exposure rate may be higher among lower grade students [19].

Third, female students maintain closer contact with their parents, and this includes conversing more with them. According to the 2014 Comprehensive Survey of Korean Youth, activities with parents such as conversation and leisure time spent were higher among female students, and they also had more weekly conversation time with their fathers, who are the main source of SHS [24]. This finding is different from those of other countries, which found that students feel closer to their same-sex parents [25]. This partly explains the gender-specific home exposure depending on the sociocultural environment. As a result, it is suggested that Korean female students are more exposed to home SHS because they have closer relationships with their fathers.

It is more difficult for adolescents than adults to avoid SHS exposure in the environment, and it is necessary for them to recognize SHS exposure to voluntarily avoid it. However, according to the Florida Youth Cohort Study, the 13–14 year olds’ self-reporting concerning SHS exposure was only half that of the 17 year olds, and the degree of home SHS exposure awareness was lower at a younger age [26]. Considering these facts, since home SHS can be underestimated for those of a younger age, the actual age-specific difference is predicted to be much bigger than our results indicated.

“Protect people from tobacco smoke” in MPOWER, WHO’s policy package in response to the tobacco epidemic, proposes a policy measure to prevent SHS exposure in public spaces through an institutional regulation, with the goal of establishing completely smoke-free environments in all indoor public places and workplaces [27]. WHO Framework Convention on Tobacco Control (FCTC) Article 8 also addresses SHS, and the average implementation rate of substantive articles by FCTC parties increased from 78% in 2012 to 84% in 2014. Although there is a degree of difference for each substantive article, SHS regulation is expected to be strengthened, along with the worldwide trend toward tobacco regulation [28]. Considering that the SHS work exposure level of non-smoking adults is high (48.3%) and has continuously increased in Korea since 2005 [19], a SHS reduction effect can be expected if the regulation on public smoking is strengthened. However, since home is also a major location for SHS exposure [1], it is difficult to achieve a completely smoke-free environment through the tobacco regulation policy at public spaces alone. Especially considering the fact that home is the major location of SHS exposure for vulnerable adolescents, they will likely be continuously exposed to SHS if no measures are taken for home SHS exposure. Furthermore, SHS exposure entails another issue: it can lead to smoking initiation among adolescents [2].

The complete or partial implementation rate of the smoking ban at indoor public spaces such as workplaces, public transportation, restaurants, pubs, and bars by the FCTC parties hovered above 75% as of 2014, but the implementation rate in private vehicles was low, at below 30%, which is similar to the current smoking ban status concerning homes [28]. SHS legislative regulation in private spaces such as homes and private vehicles is difficult, in reality. Nonetheless, since the U.S. Department of Housing and Urban Development strongly encouraged the Public Housing Authority to implement a partial or full no-smoking policy in all public housing units in 2009 [29], the interest in the home SHS ban is increasing. In addition, awareness of the effects of home smoking is improving, as some US homes have smoke-free home rules in which smoking is banned regardless of time and space, and the implementation of this ban almost doubled from 43% in 1992–1993 to 83.0% in 2010–2011. The fact that ban rules increased from 9.6% to 46.1%, even in homes with smokers, marks a significant change [30]. Though non-smoking parents and home smoking restrictions are effective for protecting children from home SHS exposure separately, the effect can be maximized when both are implemented simultaneously [31]. Therefore, it is necessary to prepare protection measures for children and female students who are frequently exposed and more vulnerable to SHS through a policy, and family members should also improve their awareness of home SHS and expand smoking restriction rules.

This study is marked by the following limitations. First, the KYRBS used in this study did not survey the specific sources of home SHS. About 9% of the adolescents were exposed to SHS even when there were no smokers in the home, and the age-dependent SHS exposure pattern was the same. Therefore, it is necessary to prepare a measure to address this issue by conducting a follow-up study considering specific sources. Second, self-reporting of home SHS exposure experiences might be inaccurate. However, considering the fact that self-reported SHS is under-reported by the younger students, the differences in age-specific SHS exposure may be much bigger [26], which further supports the results of this study. Third, the SHS exposure of the female students may have been higher because they are more sensitive to SHS exposure [26]. Fourth, we cannot consider the unmeasured confounding variables such as parental characteristics (education, age,), family characteristics (family size, family structure), and parental smoking characteristics (smoking behavior and attitudes).

However, this study included a large number of non-smoking middle and high school students—over 60,000—using representative data from Korean adolescents. Since we calculated weighted statistics to represent general population of Korean adolescent, this result can be generalized to Korean adolescents. Therefore, considering that the smoking initiation rate among middle and high school students rapidly increases each year [10], we were able to evaluate the age-specific SHS exposure levels by categorizing them into 1 grade units to assess the risk factor of smoking initiation. Therefore, this result can be used not only in identifying the group most vulnerable to home SHS but also as basic data for future studies on SHS exposure and smoking initiation risks. Furthermore, it is significant that we presented results contradictory to those of existing Asian studies by analyzing Korean middle and high school students for a study on gender- and age-specific SHS exposure in Asia using students with a high likelihood of smoking initiation.

## 5. Conclusions

Girls and younger adolescents, populations vulnerable to smoke exposure, were more likely to be exposed to SHS at home, even though they should be more protected. Therefore, it is necessary to improve home SHS awareness, especially among this vulnerable population. In addition, home smoking restrictions need to be negotiated. At the same time, it is necessary to reduce SHS exposure by educating adolescents on how to avoid or reject SHS exposure at home.

## Figures and Tables

**Table 1:** Prevalence of exposure to second-hand smoke at home.

Characteristics	Total	Sex	
		Boys	Girls
Respondents	64,499	30,925 (49.3 ^a^)	33,574 (50.7 ^a^)
Sex			
Boys	9780 (31.1)		
Girls	11,682 (34.1)		
Grade			
7th	4000 (35.4)	1964 (33.5)	2036 (37.4)
8th	3912 (33.1)	1888 (31.7)	2024 (34.5)
9th	3750 (32.9)	1668 (30.3)	2082 (35.4)
10th	3289 (31.4)	1508 (30.1)	1781 (32.6)
11th	3369 (32.0)	1426 (31.0)	1913 (32.9)
12th	3142 (31.2)	1296 (30.0)	1846 (32.3)
Location			
Metropolitan city	10,676 (31.5)	4847 (30.0)	5829 (33.0)
City	9603 (33.6)	4330 (32.0)	5273 (35.0)
Province	1183 (37.4)	603 (36.4)	580 (38.3)
Perceived economic status			
High	6156 (27.7)	3188 (27.2)	2968 (28.3)
Middle	10,870 (33.8)	4738 (32.5)	6132 (35.0)
Low	4436 (39.3)	1854 (36.3)	2582 (41.9)
Closest friend smoking			
None	11,555 (29.0)	3875 (26.0)	7680 (30.8)
Some	8350 (36.7)	4827 (34.0)	3523 (41.5)
Most/All	1557 (44.3)	1078 (41.9)	479 (51.5)
Current household member smoking			
No	2443 (8.9)	1383 (10.0)	1060 (7.6)
Yes	19,019 (50.4)	8397 (47.9)	10,622 (52.6)

Data were presented as unweighted numbers (weighted percentages); ^a^ Percentages presented represent the respondents’ sexual distribution.

**Table 2:** Adjusted odds ratios ^a^ (95% confidence interval) for exposure to second-hand smoke at home.

Characteristics	Total	Sex	
		Boys	Girls
**Sex**			
Boys	Ref		
Girls	1.26 (1.21–1.32)		
Grade			
7th	1.40 (1.31–1.51)	1.37 (1.24–1.52)	1.44 (1.30–1.59)
8th	1.20 (1.12–1.28)	1.18 (1.07–1.30)	1.21 (1.10–1.34)
9th	1.14 (1.06–1.22)	1.05 (0.95–1.16)	1.23 (1.11–1.35)
10th	1.01 (0.94–1.08)	0.98 (0.89–1.09)	1.04 (0.95–1.14)
11th	1.04 (0.97–1.11)	1.05 (0.96–1.15)	1.02 (0.93–1.12)
12th	Ref	Ref	Ref
*p* for trend	<0.001	<0.001	<0.001
Location			
Metropolitan city	Ref	Ref	Ref
City	1.07 (1.03–1.12)	1.06 (0.99–1.12)	1.08 (1.02–1.15)
Province	1.21 (1.09–1.34)	1.26 (1.11–1.43)	1.16 (0.98–1.37)
*p* for trend	<0.001	0.002	0.003
Perceived economic status			
High	Ref	Ref	Ref
Middle	1.19 (1.14–1.24)	1.12 (1.09–1.19)	1.27 (1.20–1.35)
Low	1.41 (1.33–1.50)	1.29 (1.19–1.39)	1.55 (1.44–1.68)
*p* for trend	<0.001	<0.001	<0.001
Closest friend smoking			
None	Ref	Ref	Ref
Some	1.54 (1.48–1.61)	1.52 (1.43–1.61)	1.57 (1.48–1.66)
Most/All	2.21 (2.04–2.41)	2.12 (1.92–2.35)	2.43 (2.04–2.90)
*p* for trend	<0.001	<0.001	<0.001
Current household member smoking			
No	Ref	Ref	Ref
Yes	9.98 (9.51–10.47)	7.91 (7.41–8.44)	12.89 (12.05–13.78)

^a^ Adjusted for all other variables.

**Table 3:** The current household member smoker-stratified adjusted odds ratios ^a^ (95% confidence interval) for exposure to second-hand smoke at home according to grade by sex.

Sex	Stratification		Grade						
			12th	11th	10th	9th	8th	7th	*p* for Trend
Boys	Current household member smoking	No	Ref	1.13 (0.94–1.35)	0.77 (0.63–0.93)	0.94 (0.77–1.15)	0.97 (0.79–1.18)	1.31 (1.06–1.61)	0.321
		Yes	Ref	1.03 (0.93–1.14)	1.06 (0.95–1.19)	1.09 (0.98–1.22)	1.25 (1.11–1.39)	1.39 (1.24–1.57)	<0.001
Girls	Current household member smoking	No	Ref	1.01 (0.84–1.21)	1.04 (0.86–1.27)	1.09 (0.88–1.34)	1.28 (1.02–1.59)	1.33 (1.07–1.66)	0.001
		Yes	Ref	1.03 (0.94–1.13)	1.04 (0.94–1.14)	1.26 (1.14–1.39)	1.20 (1.08–1.33)	1.46 (1.31–1.62)	<0.001

^a^ Adjusted for location, perceived economic status, and closest friend smoking.

**Table 4:** The current household smoker-stratified adjusted odds ratios ^a^ (95% confidence interval) for exposure to second-hand smoke at home according to sex by grade.

Stratification by Current Household Smoker	Sex	
	Boys	Girls
No		
7th	Ref	0.82 (0.67–1.01)
8th	Ref	0.98 (0.77–1.24)
9th	Ref	0.96 (0.76–1.20)
10th	Ref	1.29 (1.03–1.61)
11th	Ref	0.87 (0.72–1.05)
12th	Ref	1.00(0.82–1.23)
Total	Ref	0.96(0.88–1.05)
Yes		
7th	Ref	1.34 (1.21–1.48)
8th	Ref	1.24 (1.12–1.37)
9th	Ref	1.52 (1.38–1.67)
10th	Ref	1.29 (1.15–1.44)
11th	Ref	1.42 (1.27–1.60)
12th	Ref	1.42 (1.25–1.61)
Total	Ref	1.35 (1.29–1.42)

^a^ Adjusted for location, perceived economic status, and closest friend smoking.

## Supplementary Materials

The following are available online at www.mdpi.com/1660-4601/13/2/241/s1, Table S1. The original Korean version of core questionnaires.
