# Supplementary material for: Sex and Age Differences in Exposure to Secondhand Smoke at Home among Korean Adolescents: A Nationally Representative Survey
Source: Int J Environ Res Public Health. 2016 Feb 19;13(2):241. doi: 10.3390/ijerph13020241 (PMC4772261; doi:10.3390/ijerph13020241)
Supplement: Supplementary file 1 [file ijerph-13-00241-s001.pdf]

Supplementary Materials: Sex and Age Differences in Exposure to Secondhand Smoke at Home among Korean Adolescents: A Nationally Representative Survey

Jun Hyun Hwang and Soon-Woo Park

Table S1. The original Korean version of core questionnaires.

English Version	Korean Version
Q1. During the past 7 days, on how many days have people smoked in your home, in your presence?	Q1. 최근 7 일 동안, 학생의 집안에서 다른 사람(가족이나 손님 등)이 담배를 피울 때 그 근처에 같이 있은 적이 며칠입니까?
① 0 day	① 최근 7 일 동안 없다
② 1 day	② 주 1 일
③ 2 days	③ 주 2 일
④ 3 days	④ 주 3 일
⑤ 4 days	⑤ 주 4 일
⑥ 5 days	⑥ 주 5 일
⑦ 6 days	⑦ 주 6 일
⑧ 7 days	⑧ 매일
Q2. Please indicate all the current smoker in your family	Q2. 가족 중에서 현재 담배를 피우시는 분은 모두 표시해 주십시오.
① None	①없다
② Father	② 아버지
③ Mother	③ 어머니
④ Siblings	④ 형제 자매
⑤ Grandparents	⑤ 할아버지 할머니
⑥ Others	⑥ 기타
⑦ Don't Know	⑦ 잘 모른다
Q3. Do any of your closest friends smoke tobacco?	Q3. 친한 친구 중에 담배를 피우는 친구가 있습니까?
① None of them	① 아무도 안 피운다
② Some of them	② 몇 명은 담배를 피운다
③ Most of them	③ 대부분은 담배를 피운다
④ All of them	④ 모두 담배를 피운다

© 2016 by the authors; licensee MDPI, Basel, Switzerland. This article is an open access article distributed under the terms and conditions of the Creative Commons by Attribution (CC-BY) license (http://creativecommons.org/licenses/by/4.0/).
